# Supplementary material for: SLC38A5 Modulates Ferroptosis to Overcome Gemcitabine Resistance in Pancreatic Cancer
Source: Cells. 2023 Oct 23;12(20):2509. doi: 10.3390/cells12202509 (PMC10605569; doi:10.3390/cells12202509)
Supplement: Supplementary file 1 [file cells-12-02509-s001.zip › cells-2633587-supplementary.pdf]

Supplementary figure S1

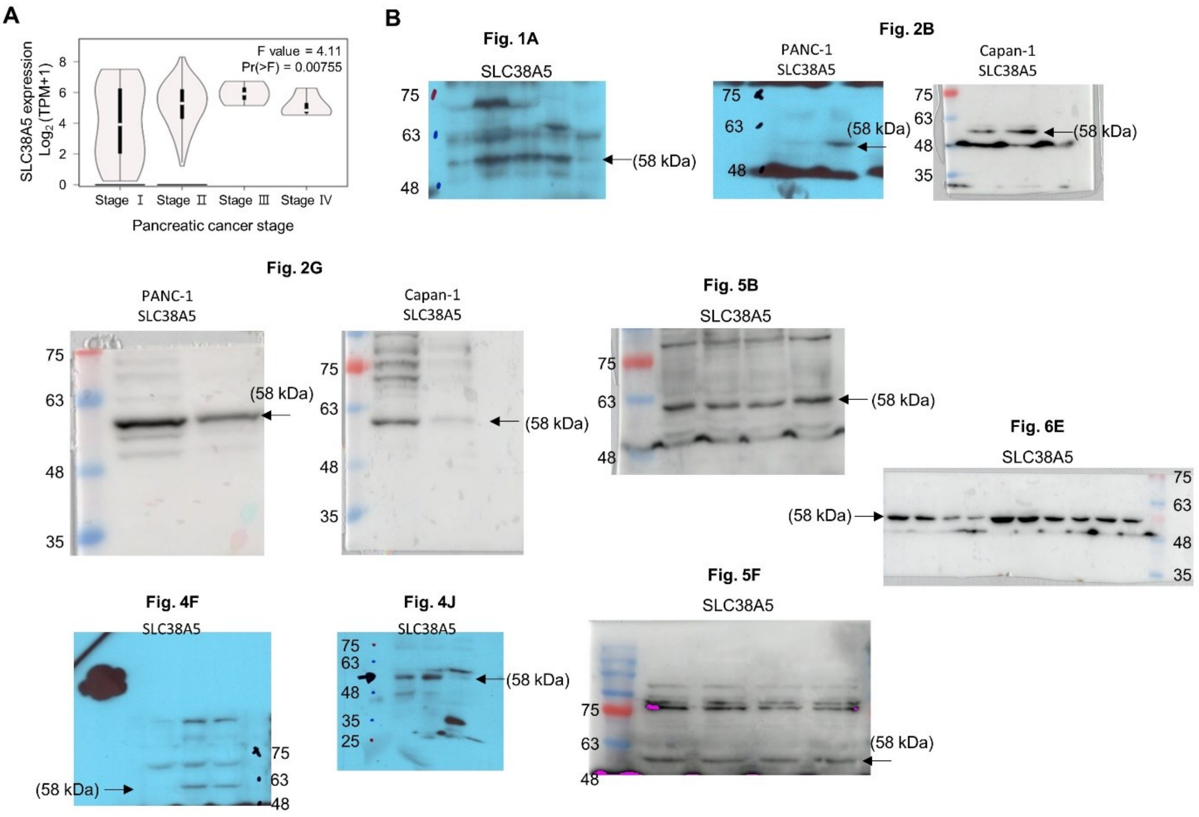

Supplementary Figure S1. The stage plot of SLC38A5 in pancreatic cancer and expression of SLC38A5

Supplementary figure S2

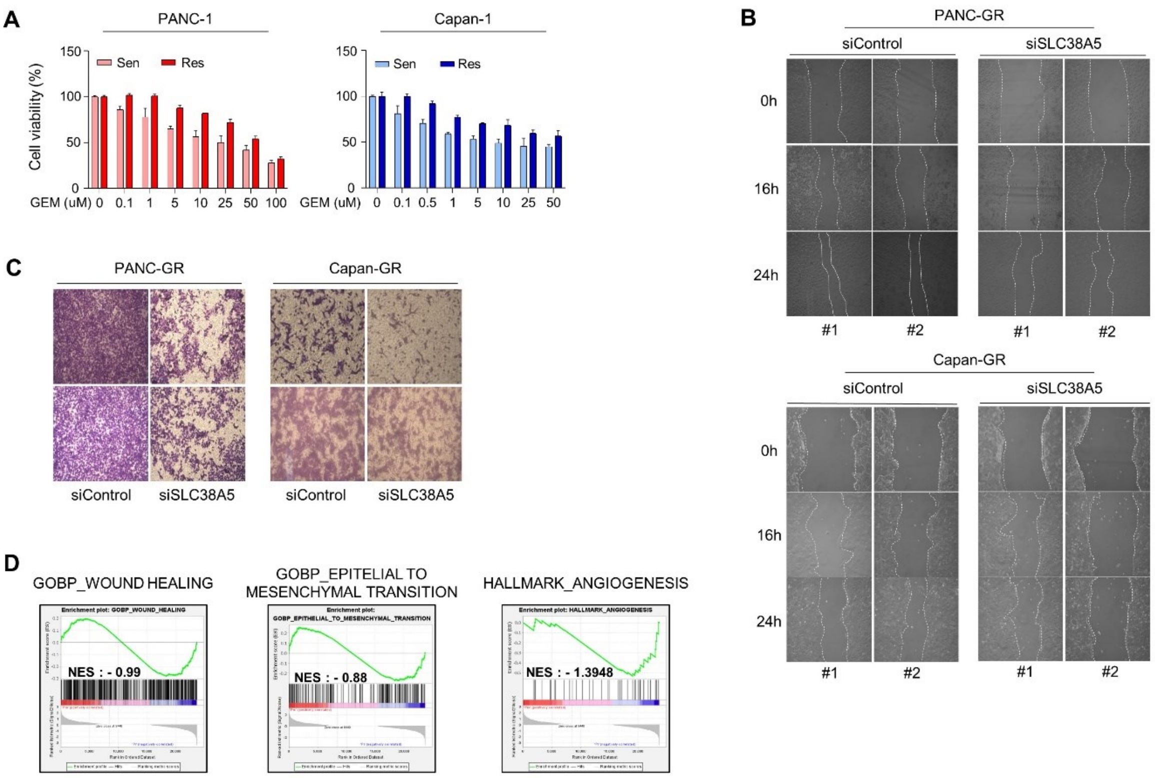

Supplementary Figure S2. Deletion of SLC38A5 down-regulates cell viability and migration in gemcitabine-resistance pancreatic cancer cells. (A) WST assay is performed to determine cell viability for gemcitabine in PANC-1 and Capan-1. (B) Two additional wound healing experiments were conducted to obtain the standard deviation for cell migration. (C) Two additional transwell invasion assay were conducted to obtain the standard deviation for cell invasiveness. (D) GSEA for significant enrichment for wound healing, EMT and angiogenesis in PANC-GR.

Supplementary figure S3

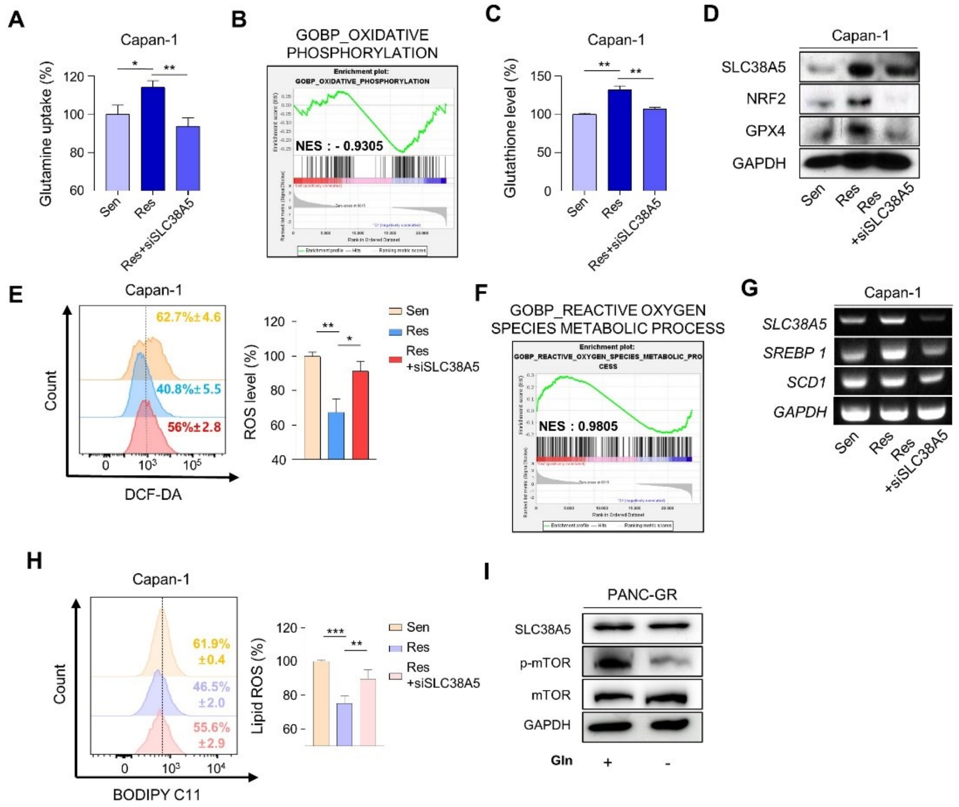

Supplementary Figure S3. SLC38A5 modulates lipid ROS through GHS-mediated ROS and mTOR-SREBP1 signaling in gemcitabine-resistance pancreatic cancer cells. (A) Comparison of glutamine uptake of Capan-1, Capan-GR and Capan-GR with siSLC38A5. (B) GSEA for significant enrichment for oxidative phosphorylation. (C) Comparison of glutathione level of Capan-1, Capan-GR and Capan-GR with siSLC38A5. (D) Expression of glutathione-related genes in Capan-1, Capan-GR and Capan-GR with siSLC38A5 is analyzed by western blot. (E) Relative ROS production in Capan-1, Capan-GR and Capan-GR with siSLC38A5 were measured using flow cytometry after dichlorofluorescein (DCF)-staining. Bar graph was analyzed by Image J software. (F) GSEA for significant enrichment for ROS level. (G) Expression of SREBP1-SCD1 signaling genes were analyzed by RT-PCR. (H) Flow cytometry analysis of lipid ROS in Capan-1, Capan-GR and Capan-GR with siSLC38A5. Bar graph is analyzed by Image J software. (I) Comparison of phospho-mTOR expression in the absence of glutamine in PANC-GR. Bar represent means ± SD. *, $P < 0.05$; **, $P < 0.01$; ***, $P < 0.001$.
